# Vulvar Dermatofibrosarcoma Protuberans in a Postmenopausal Woman: A Rare Case Report and Insights Into Treatment

**DOI:** 10.7759/cureus.118959

**Published:** 2026-11-01

**Authors:** Naw Phu Ngone Ko Ko, Ei Ei Phyo Naing

**Affiliations:** 1 Obstetrics and Gynaecology, Queen Elizabeth Hospital King's Lynn NHS Foundation Trust, King's Lynn, GBR; 2 Obstetrics and Gynaecology, Luton and Dunstable University Hospital NHS Foundation Trust, Luton, GBR

**Keywords:** gynae oncology, gynae surgeries, immunohistochemistry (cd34, vulvar dfsp, vulvar tumour

## Abstract

Dermatofibrosarcoma protuberans (DFSP) is a rare cutaneous fibroblastic neoplasm characterized by locally infiltrative growth, a tendency for local recurrence, and low metastatic potential. Vulvar involvement is exceptionally uncommon and presents diagnostic and therapeutic challenges because of both its rarity and the anatomical importance of preserving vulvar function while obtaining adequate oncological clearance. We report the case of a 56-year-old postmenopausal woman, para 4, with a BMI of 32 kg/m², who presented with a 2 × 3 cm non-tender, freely mobile lesion of the left vulva. Excisional biopsy demonstrated a proliferation of spindle cells in a characteristic storiform pattern, with diffuse CD34 immunoreactivity, confirming DFSP. The closest histological margin following the initial excision was 0.3 cm. The patient was subsequently referred to a gynaecological oncologist, and following a multidisciplinary discussion, she underwent wide local re-excision with bilateral inguinofemoral lymphadenectomy. Histopathological examination demonstrated clear surgical margins, while all 13 lymph nodes showed reactive changes without evidence of tumour involvement. The case was discussed with a radiation oncologist, and adjuvant radiotherapy was not recommended after complete surgical excision and in the absence of residual or regional disease. No systemic therapy was administered. The patient remained free of documented recurrence for more than three years. This case highlights the importance of considering DFSP in the differential diagnosis of unusual vulvar masses, obtaining histopathological confirmation, and achieving complete excision with negative margins. It also illustrates the value of multidisciplinary management in rare vulvar soft-tissue tumours.

## Introduction

Dermatofibrosarcoma protuberans (DFSP) is an uncommon cutaneous fibroblastic tumour characterized by slow growth, local infiltration, and a tendency for local recurrence after inadequate excision. Although locally aggressive, conventional DFSP has a low propensity for metastatic spread [1,2]. Clinically, vulvar DFSP may resemble more common benign vulvar lesions because of its slow growth and relatively non-specific clinical appearance. Reported cases have demonstrated the potential for initial misclassification, including misclassification as other benign or mesenchymal vulvar lesions [3-5]. Histopathological assessment is therefore essential for establishing the diagnosis. Histologically, the tumour classically consists of relatively uniform spindle cells arranged in a storiform or cartwheel pattern, and tumour cells commonly demonstrate diffuse CD34 immunoreactivity [1]. DFSP most frequently affects the trunk and proximal extremities, while vulvar involvement is exceptionally uncommon. A literature review published in 2017 identified 54 patients with vulvar DFSP [3], and a subsequent systematic review by the MITO Rare Cancer Group identified 68 reported cases from 39 publications [4]. A more recent review analysing 85 vulvar cases further emphasized the potential for diagnostic delay and initial misclassification of these lesions [5]. Complete surgical excision with histologically negative margins remains the principal treatment [2,6]. Contemporary European guidance recommends micrographically controlled surgery where available, while conventional excision with approximately 2-3 cm lateral safety margins remains an acceptable alternative when standard histopathological margin assessment is used [2]. We report a rare case of vulvar DFSP in a postmenopausal woman treated by initial excision followed by wider re-excision and discuss the diagnostic and therapeutic considerations relevant to this uncommon presentation. This case illustrates important management considerations, particularly the need to achieve adequate surgical margins in an infiltrative tumour and the limited role of elective regional lymph-node surgery in conventional DFSP, in which lymphatic metastasis is uncommon.

An earlier version of this case was presented as an electronic poster at the RCOG World Congress, London, in 2025.

## Case presentation

A 56-year-old postmenopausal woman, para 4, with a BMI of 32 kg/m², presented with a lump affecting the left vulva. Clinical examination demonstrated a non-tender, freely mobile lesion measuring approximately 2 × 3 cm and approximately 1 cm in thickness. The lesion was situated approximately 2 cm from the vaginal opening. The overlying skin was described as thin and vascularised. The uterus and cervix were atrophic on examination, and subsequent liquid-based cervical cytology was normal.

An excisional biopsy was performed. Histopathological examination demonstrated a proliferation of spindle-shaped cells arranged in a characteristic storiform pattern (Figures 1A-1F). Immunohistochemical staining demonstrated diffuse CD34 positivity in the lesional cells (Figures 2A-2E), supporting a diagnosis of dermatofibrosarcoma protuberans. The closest histological margin following the initial excision measured 0.3 cm. In view of the infiltrative nature of DFSP and the narrow margin, the patient was referred to a gynaecological oncologist for further management.

**Figure 1:**
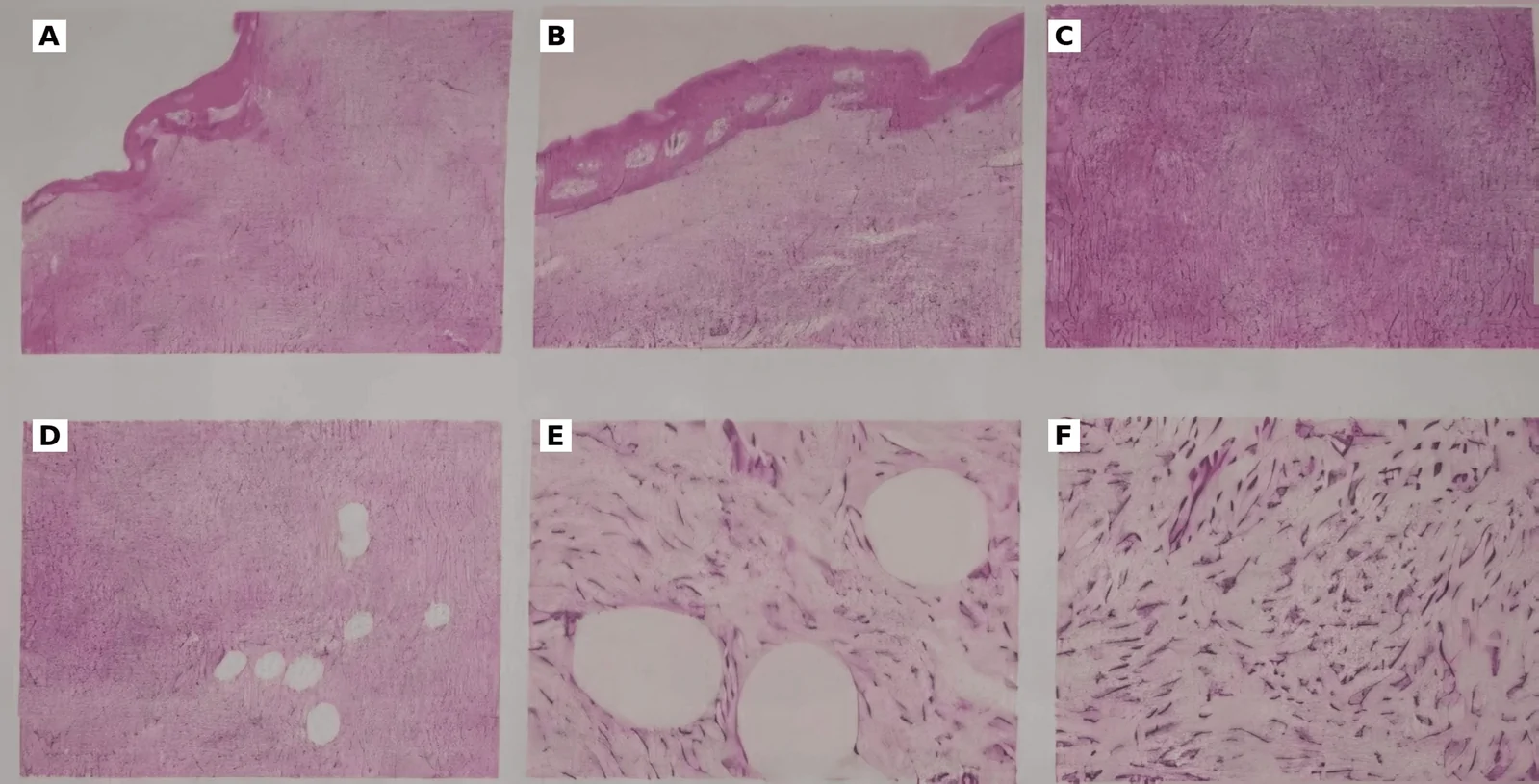
Histopathological features of the initial vulvar lesion. (A-F) H&E-stained sections at varying magnifications showing a spindle-cell tumour with a characteristic cartwheel (storiform) pattern. Mild to moderate nuclear pleomorphism and occasional mitotic activity are present, with lesional spindle cells extending into the dermis and adipose tissue.

**Figure 2:**
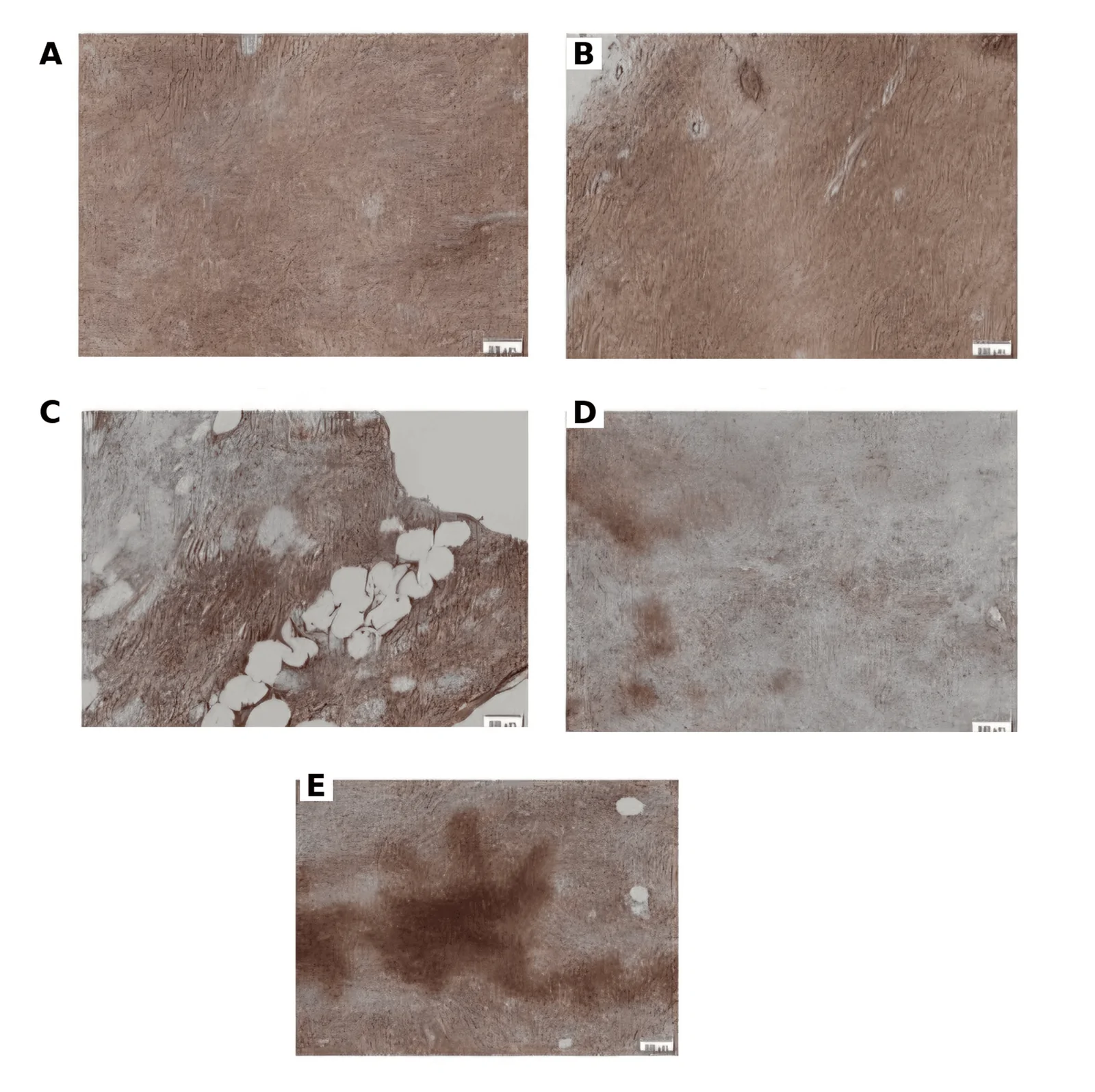
CD34 immunohistochemical features of the initial vulvar lesion. (A-E) CD34 immunohistochemical staining at varying magnifications demonstrating diffuse positivity in the lesional cells, supporting the diagnosis of dermatofibrosarcoma protuberans.

Following a multidisciplinary discussion, re-excision was performed one month later by the gynaecological oncologist. The patient underwent wide local excision together with bilateral inguinofemoral lymphadenectomy. Histopathological examination of the re-excision specimen demonstrated a 4-mm focus of residual dermatofibrosarcoma protuberans with clear surgical margins (Figures 3A-3F). Thirteen excised lymph nodes showed reactive changes with no evidence of tumour involvement. However, the exact lateral and deep clearance distances were not recorded in the available pathology report. The precise rationale for performing bilateral inguinofemoral lymphadenectomy is not documented in the source material available for this report. The procedure formed part of the management undertaken at the treating institution at that time.

**Figure 3:**
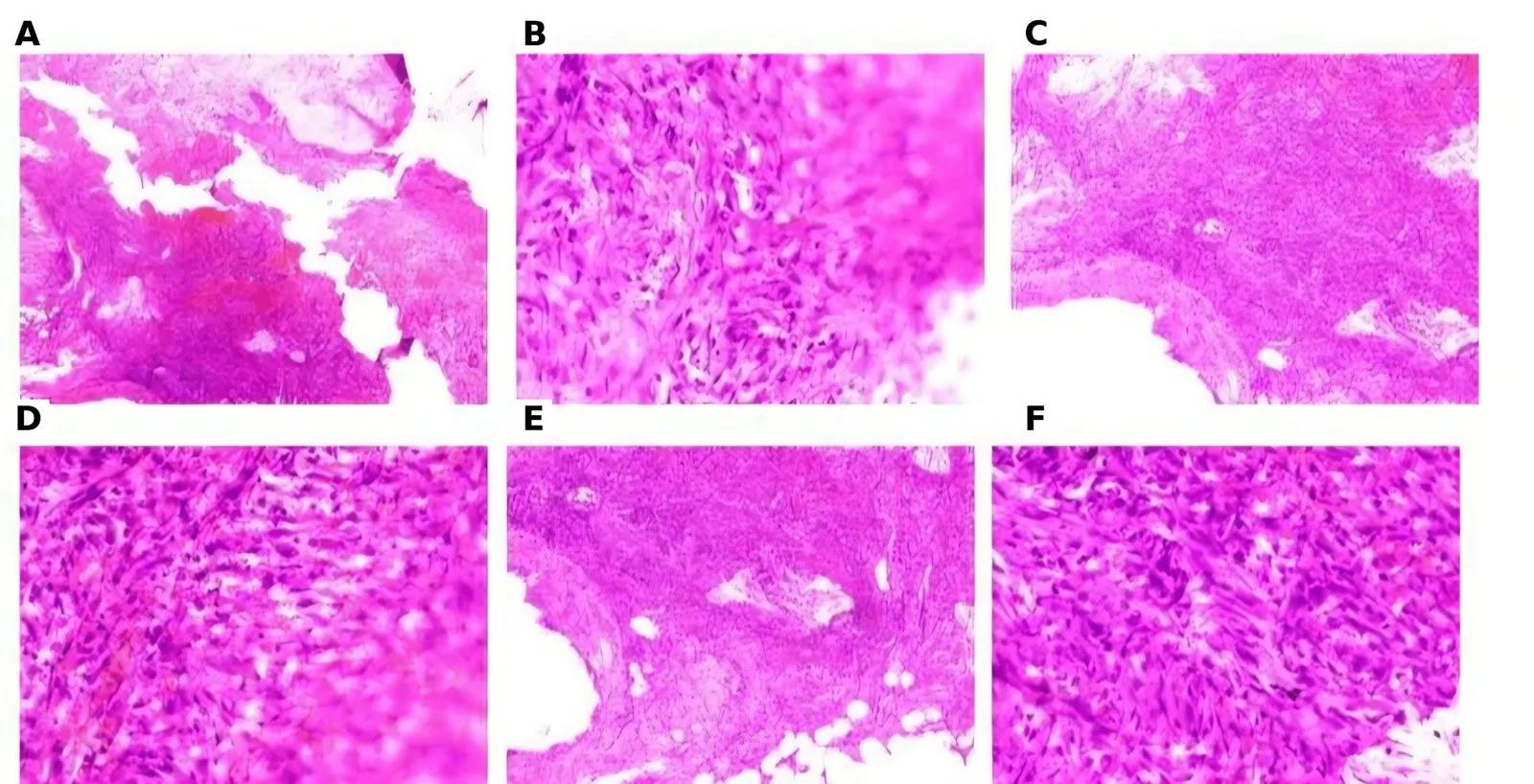
Histopathological features of the re-excision specimen. (A-F) H&E-stained sections at varying magnifications demonstrating a residual spindle-cell tumour composed of proliferating spindle cells arranged in a characteristic cartwheel (storiform) pattern. Mild to moderate nuclear pleomorphism and occasional mitotic activity are present, with lesional spindle cells extending into the dermis and adipose tissue. All resection margins were free of lesional cells.

The case was subsequently discussed with a radiation oncologist as part of multidisciplinary management. Given the complete surgical excision with clear margins and no evidence of remaining residual or regional disease, adjuvant radiotherapy was not recommended. No systemic therapy was administered.

No short- or long-term postoperative complications following bilateral inguinofemoral lymphadenectomy were reported, including lower-limb lymphoedema, chronic wound infection, lymphocele, or neuropathic pain. The patient was reviewed every three months during the first year and every six months during the subsequent two years, with annual follow-up planned thereafter. Clinical examination and ultrasonography were performed at each follow-up visit. Based on the available follow-up information, the patient remained free of documented recurrence for more than three years.

No preoperative cross-sectional imaging was performed before either the initial excision or the subsequent re-excision. The case was managed in Myanmar in 2022 in a resource-limited setting during a period of significant disruption to healthcare services, and access to advanced imaging was constrained. Surgical planning was therefore guided by clinical examination.

## Discussion

Histopathological and molecular diagnosis

Conventional DFSP typically consists of relatively uniform spindle cells arranged in a storiform pattern. Diffuse CD34 positivity is characteristic and provides valuable diagnostic support [1]. The characteristic molecular abnormality in most cases is a t(17;22) translocation resulting in a COL1A1-PDGFB fusion [1,7]. In this patient, the combination of spindle-cell proliferation, storiform architecture, and diffuse CD34 immunoreactivity supported the diagnosis. Molecular testing for the COL1A1-PDGFB fusion was not documented in the available case material. Assessment for fibrosarcomatous transformation is important because fibrosarcomatous DFSP is associated with more aggressive clinical behaviour, including higher risks of local recurrence and metastatic disease [1,2,5]. In this case, the available pathology documentation did not specifically state whether fibrosarcomatous transformation was present or absent.

Surgical management

Complete surgical excision with negative histological margins remains the cornerstone of treatment. The 2025 European interdisciplinary guideline recommends micrographically controlled surgery as the preferred surgical approach because of the infiltrative growth pattern of DFSP. Conventional excision with a lateral safety margin of approximately 2-3 cm is an acceptable alternative when micrographic surgery is not available [2]. The vulva presents a particular surgical challenge because wide excision may result in functional, cosmetic, sexual, and wound-healing morbidity. Tissue-preserving approaches that allow comprehensive margin assessment may therefore be particularly advantageous at this anatomical site. Mohs or slow-Mohs techniques have been successfully used for genital DFSP and may allow maximal preservation of healthy tissue while maintaining oncological clearance [3,8]. In the present case, the closest histological margin following the initial excision was 0.3 cm. Given the infiltrative nature of DFSP and the narrow margin, further surgery was undertaken. Re-excision subsequently achieved histologically clear margins. Alshaygy I et al. reported outcomes from a large surgical series and demonstrated excellent local control when adequate resection was achieved, further supporting the importance of obtaining negative surgical margins [9].

Role of regional lymph-node surgery

The patient underwent bilateral inguinofemoral lymphadenectomy, with all 13 lymph nodes showing reactive changes and no evidence of tumour involvement. The use of bilateral inguinofemoral lymphadenectomy in this case should be considered in the context of the low risk of lymphatic spread in conventional DFSP. Regional lymph-node metastasis is rare. A large historical review of 913 cases reported regional lymph-node metastases in approximately 1% of patients and concluded that elective lymph-node dissection was not recommended [10]. Subsequent literature has continued to describe regional lymph-node involvement as exceptionally uncommon [11]. Although bilateral inguinofemoral lymphadenectomy formed part of this patient's treatment at the time, routine elective lymphadenectomy is not generally considered standard management for clinically node-negative conventional DFSP. The principal oncological objective remains complete local tumour excision with negative margins. Regional nodal surgery may be considered when there is clinical, radiological, or pathological evidence of lymph-node involvement; however, such circumstances are uncommon, and management should be individualised within a multidisciplinary setting [10,11].

Role of radiotherapy and systemic therapy

Following re-excision, the case was discussed with a radiation oncologist as part of the multidisciplinary management process. As complete surgical clearance had been achieved and there was no evidence of residual or regional disease, adjuvant radiotherapy was not recommended. This approach is consistent with contemporary management principles. Current NCCN guidance emphasises complete surgical resection and considers radiotherapy or systemic therapy in selected circumstances where adequate surgical clearance cannot be achieved or where the disease is unresectable [12]. The European guideline similarly identifies surgery as the principal treatment and recognises imatinib as an option for inoperable primary tumours, locally inoperable recurrence, and metastatic DFSP [2]. Imatinib targets the platelet-derived growth factor receptor pathway associated with the COL1A1-PDGFB fusion and may also be considered selectively for large or difficult-to-resect tumours to facilitate subsequent surgery. No radiotherapy or systemic therapy was administered following complete surgical excision in this patient.

Follow-up and prognosis

The patient remained free of documented recurrence for more than three years. Long-term clinical surveillance is important because DFSP can recur even after apparently adequate treatment. Huis In 't Veld EA et al. evaluated 357 patients treated at three tertiary centres and reported that clinical follow-up for up to 10 years has traditionally been recommended, although surveillance intensity may be individualised according to recurrence risk [13]. A more recent review of vulvar DFSP found that most recurrences occurred within the first three years following wide excision and recommended regular follow-up [5]. Follow-up should include clinical assessment of the operative site and patient education regarding new or recurrent vulvar lesions. More intensive surveillance may be appropriate in patients with fibrosarcomatous transformation, positive or close margins, recurrent disease, or other adverse pathological features.

## Conclusions

Vulvar DFSP is an exceptionally rare cutaneous soft-tissue tumour that may initially resemble a benign vulvar mass. Histopathological examination remains essential for diagnosis, with spindle-cell proliferation in a storiform pattern and diffuse CD34 immunoreactivity providing characteristic diagnostic features. Complete surgical excision with histologically negative margins remains the cornerstone of treatment. Micrographically controlled surgery offers the advantage of comprehensive margin assessment with maximal tissue preservation, although appropriately performed wide local excision remains an acceptable alternative. This case also highlights an important management consideration: routine elective regional lymphadenectomy is not generally indicated in clinically node-negative conventional DFSP because lymphatic metastasis is uncommon. The favourable postoperative outcome in this patient should therefore not be interpreted as evidence that regional lymphadenectomy was necessary or beneficial. Multidisciplinary discussion is valuable in determining whether further surgery, radiotherapy, or systemic therapy is required. Continued clinical surveillance is appropriate because local recurrence may occur even after apparently complete excision. Likewise, the absence of documented recurrence during more than three years of follow-up reflects the observed course of this individual case and does not establish definitive long-term disease control.
